# Pegylated Gold Nanoparticles Induce Apoptosis in Human Chronic Myeloid Leukemia Cells

**DOI:** 10.1155/2014/182353

**Published:** 2014-03-25

**Authors:** Yu-Chuen Huang, Yuh-Cheng Yang, Kai-Chien Yang, Hui-Ru Shieh, Tao-Yeuan Wang, Yeukuang Hwu, Yu-Jen Chen

**Affiliations:** ^1^Department of Medical Research, China Medical University Hospital, Taichung 404, Taiwan; ^2^School of Chinese Medicine, China Medical University, Taichung 404, Taiwan; ^3^Department of Gynecology and Obstetrics, Mackay Memorial Hospital, Taipei 104, Taiwan; ^4^Department of Medical Research, Mackay Memorial Hospital, Taipei 104, Taiwan; ^5^Department of Pathology, Mackay Memorial Hospital, Taipei 104, Taiwan; ^6^Institute of Physics, Academia Sinica, 128 Academia Road, Section 2, Taipei 115, Taiwan; ^7^Department of Radiation Oncology, Mackay Memorial Hospital, 92 Chung San North Road, Section 2, Taipei 104, Taiwan; ^8^Institute of Traditional Medicine, National Yang-Ming University, Taipei 112, Taiwan

## Abstract

Gold nanoparticles (AuNPs) have several potential biological applications as well as excellent biocompatibility. AuNPs with surface modification using polyethylene glycol (PEG-AuNPs) can facilitate easy conjugation with various biological molecules of interest. To examine the anticancer bioactivity of PEG-AuNPs, we investigated their effect on human chronic myeloid leukemia K562 cells. The results indicated that PEG-AuNPs markedly inhibited the viability and impaired the cell membrane integrity of K562 cells. The particles caused morphological changes typical of cell death, and a marked increase in the sub-G1 population in DNA histogram, indicating apoptosis. In addition, PEG-AuNPs reduced the mitochondrial transmembrane potential, a hallmark of the involvement of intrinsic apoptotic pathway in K562 cells. Observation of ultrastructure under a transmission electron microscope revealed that the internalized PEG-AuNPs were distributed into cytoplasmic vacuoles and damaged mitochondria, and subsequently accumulated in areas surrounding the nuclear membrane. In conclusion, PEG-AuNPs may have the potential to inhibit growth and induce apoptosis in human chronic myeloid leukemia cells.

## 1. Introduction

Nanotechnology has emerged as a broad and rapidly developing field over the past few decades. The application of nanomaterials in biomedicine is currently increasing owing to growing evidence of its benefits in cancer prevention and treatment [1]. Among a variety of nanomaterials, gold nanoparticles (AuNPs) possess unique characteristics: small size, good biocompatibility, low toxicity, simple surface chemistry, and easy surface modification. These features make AuNPs very promising candidates for biomedical use and numerous biological applications as biosensors and drug delivery vectors for cancer chemotherapy and radiotherapy [2–5].

However, administration of nanoparticles to target tumor sites is usually limited owing to uptake and rapid elimination by the reticuloendothelial system, particularly macrophages. Nanoparticles coated by polyethylene glycol (PEGylation) can create a hydrophilic protective layer to prevent the absorption of opsonin proteins, thereby limiting the recognition and clearance by macrophages and prolonging their circulating lifetime [6, 7]. PEGylated nanoparticles reportedly have very low cytotoxic effect on many cell lines, such as HeLa, EMT-6, and CT26 cells [6, 8–10], which is reflected in* in vivo* studies irrespective of the route of administration [11]. PEGylation is considered as a very effective and biocompatible way to decrease renal clearance.

These benefits of PEGylation are normally realized by solution chemistry method. Recently, we reported a new approach for the synthesis of AuNPs coated with polyethylene glycol (PEG-AuNPs) [9, 10, 12–15] using very intense X-ray radiation generated by synchrotron radiation. The PEG-AuNPs produced by this approach have a uniform spherical shape with a mean diameter of 6.1 ± 1.9 nm (determined by transmission electron microscopy, TEM). In a one-pot process, AuNPs were synthesized from direct reduction of Au ions by X-rays, and the PEG were then absorbed on the AuNP surfaces without adding other chemicals. The synthesized colloidal substances are quite stable even at very high concentrations under both* in vitro* and* in vivo* conditions [9, 10, 12–15]. The PEG-AuNPs are highly biocompatible and can strongly enhance cell damage induced by X-ray irradiation [12]. Surviving cells decreased by approximately 2–45% after irradiation in the presence of PEG-AuNPs [9]. Moreover, our results demonstrated that PEG-AuNPs markedly accumulate by approximately 25-fold more in tumor tissues than in normal muscle tissue [15]. Therefore, PEG-AuNPs have valuable applications in enhancing X-ray tumor imaging and radiotherapy and have a great potential for customized therapy [14, 16].

In the present study, we used human chronic myeloid leukemia K562 cells as a model to evaluate the cytotoxicity of PEG-AuNPs in a specific cancer cell line and to elucidate the mode of cell death. This is an important step for further investigation of the possibility of employing similar treatment in cancer. Intracellular localization of PEG-AuNPs was also assessed.

## 2. Materials and Methods

### 2.1. Preparation of Gold Nanoparticles

PEG-AuNPs were synthesized using X-ray irradiation as previously reported [9, 10, 12, 14, 15, 17, 18]. Well-dispersed PEG-gold colloidal solutions were produced by irradiating a mixed aqueous solution of gold precursors and PEG (Molecular weight of 6000) in NaOH (0.1 M, Showa Inc., Japan), with very intense X-radiation generated by a synchrotron facility. The very intense X-ray radiolyzed water and the created free radicals reduced the Au ion into its precursors and formed metal clusters. PEG was absorbed on the AuNPs passive surface and further particle growth and aggregation was terminated. A uniform and instantaneous reduction occurs due to the high penetration and intensity of the synchrotron X-rays, leading to the generation of high-quality nanoparticles in well-controlled sizes. The PEG-AuNPs were spherical with a diameter of 6.1 ± 1.9 nm (determined by TEM). Since the PEG molecules used did not have any open bonding on the end of their chain structure, we assume that the PEG molecules were adsorbed to coat the Au surfaces. The average amount of PEG molecule on each AuNP was around 50. The PEG coated on the AuNP surface not only stabilized the otherwise very unstable bare AuNP but also facilitated its use in biomedical applications. The zeta potential for the unmodified gold colloid was −57.8 ± 5 mV. The zeta potential for the PEG-gold colloid decreased to −20.1 ± 7.5 mV at pH 7.23. Extensive characterization and tests were performed on these PEG-AuNPs and it was generally concluded that they are highly compatible with all the cell lines and animals.

### 2.2. Cell Lines and Cultures

Human chronic myeloid leukemia cell line K562 (ATCC, Manassas, VA, USA) was used in this study. K562 cells were cultured in RPMI 1640 (Gibco BRL, Rockville, MD, USA) and supplemented with 10% heat-inactivated fetal bovine serum (HyClone), penicillin (100 IU/mL), streptomycin (100 mg/mL), and L-glutamine (2 mM). The cells were maintained at 37°C in a humidified incubator with 5% CO_2_. The PEG-AuNPs colloidal solution was dispersed in RPMI 1640 medium and incubated with K562 cells for 24–72 h.

### 2.3. Cell Viability and Cell Membrane Integrity

K562 cells (1 × 10^5^ cells/mL) were treated with 5 or 10 mM PEG-AuNPs for 24–72 h. The numbers of viable cells were measured by trypan blue dye exclusion assay. The exclusion of trypan blue was also considered as an index of cell membrane integrity [19].

### 2.4. Morphological Examination by Light Microscope

For morphological examination, the cells were cytocentrifuged onto a microscope slide using a Cytospin^4^ (Shandon Southern Instrument Inc., Sewelicky, PA, USA), stained with Liu's stain, and observed under an upright microscope (Olympus, Tokyo, Japan) at a magnification of 1,000x.

### 2.5. Morphological Examination by TEM

Cells were washed and fixed using a cold buffer containing 3% glutaraldehyde in 0.11 M cacodylate for 30 min. After rinsing with phosphate-buffered saline (PBS), the cells were postfixed in osmium tetroxide (1%) and embedded in Epon resin. Semithin sections were cut, stained with 0.5% toluidine blue, and examined under a light microscope. Ultrathin sections were stained with 2% uranyl acetate and Reynolds lead citrate and observed with a transmission electron microscope (JEOL Co., JEM-1200EXII).

### 2.6. Cell Cycle Analysis by Flow Cytometry

After treatment, the cells were harvested and fixed with 70% ethanol at 4°C for 1 h. The cells were stained for 30 min with propidium iodide solution (propidium iodide, 0.5 mg/mL; RNase, 0.1 mg/mL) contained in a Cycletest Plus DNA reagent kit (Becton Dickinson, Lincoln Park, NJ, USA). Analysis of the DNA histogram was performed using a FACScalibur flow cytometer (Becton Dickinson). The data collected by examining 10^4^ cells were analyzed using ModFit software (Becton Dickinson).

### 2.7. Assessment of Mitochondrial Membrane Potential

After treatment, the cells were washed with PBS and incubated with 40 nM 3,3′-dihexyloxacarbocyanine [DiOC6_(3)_] (Molecular Probes, Eugene, OR, USA) for 15 min at 37°C. The fluorescence intensity was measured by FACScalibur flow cytometer (Becton Dickinson) to estimate the changes in mitochondrial transmembrane potential with excitation and emission settings of 488 and 530 nm, respectively. For the control group, the fluorescence intensity of the probe was optimized to an area within the normal distribution in the histogram to facilitate further comparison.

### 2.8. Statistical Analysis

Results are presented as mean ± standard error (SE) based on three independent experiments. One-way analysis of variance and Tukey's test were used to compare the mean cell count among different treatments. Student's *t*-test was used to compare the mean gated cells in the different cell cycle phases and relative mean value of mitochondrial transmembrane potential between the controls and cells treated with 10 mM AuNPs. Statistical analyses were performed using the SPSS software package (version 18.0) and *P* values less than 0.05 were considered significant.

## 3. Results

### 3.1. PEG-AuNPs Inhibited Growth and Impaired Cell Membrane Integrity of Human Leukemic K562 Cells

**Figure 1:**
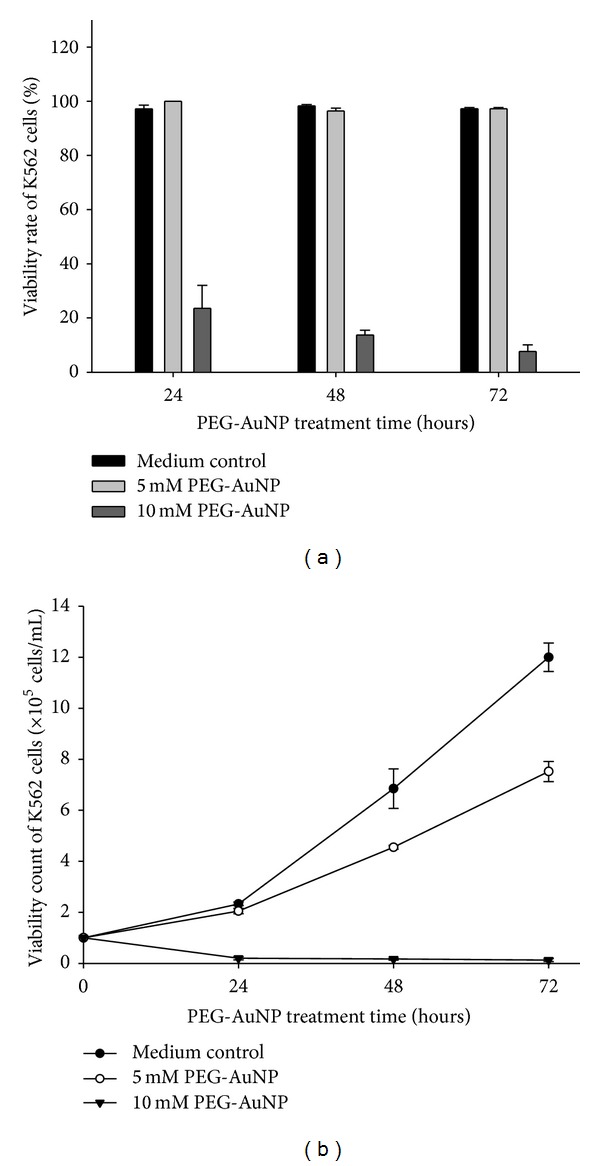
(a) Cell viability rate and (b) cell viability count of K562 cells treated with the control medium, 5 and 10 mM gold nanoparticles (AuNPs), with their surface modified by polyethylene glycol (PEG-AuNPs) for 24–72 h. Results are means ± standard error (SE) based on triplicate experiments.

To examine the effect of PEG-AuNPs on cell viability, K562 cells were treated with 5 and 10 mM PEG-AuNPs for 24, 48, and 72 h, and the viability was assessed using the trypan blue exclusion test. The results indicated that PEG-AuNPs markedly inhibited the viability of K562 cells in a dose- and time-dependent manner, particularly at 10 mM of PEG-AuNPs (Figures 1(a) and 1(b)). Since the avoidance of trypan blue could be considered as an index of cell membrane integrity, these results suggest that PEG-AuNPs could impair the cell membrane integrity.

### 3.2. Morphological Changes and Localization of PEG-AuNPs Treatment

**Figure 2:**
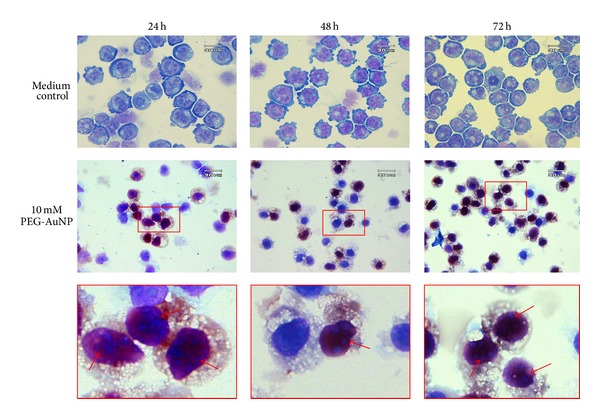
K562 cells were stained with Liu's dye for morphological examinations. Cells were treated with medium only (upper panels), 10 mM AuNPs (middle panels), after 24–72 h of treatment. The arrow indicates an apoptotic cell (lower panels). Original magnification is 1,000x for all panels.

**Figure 3:**
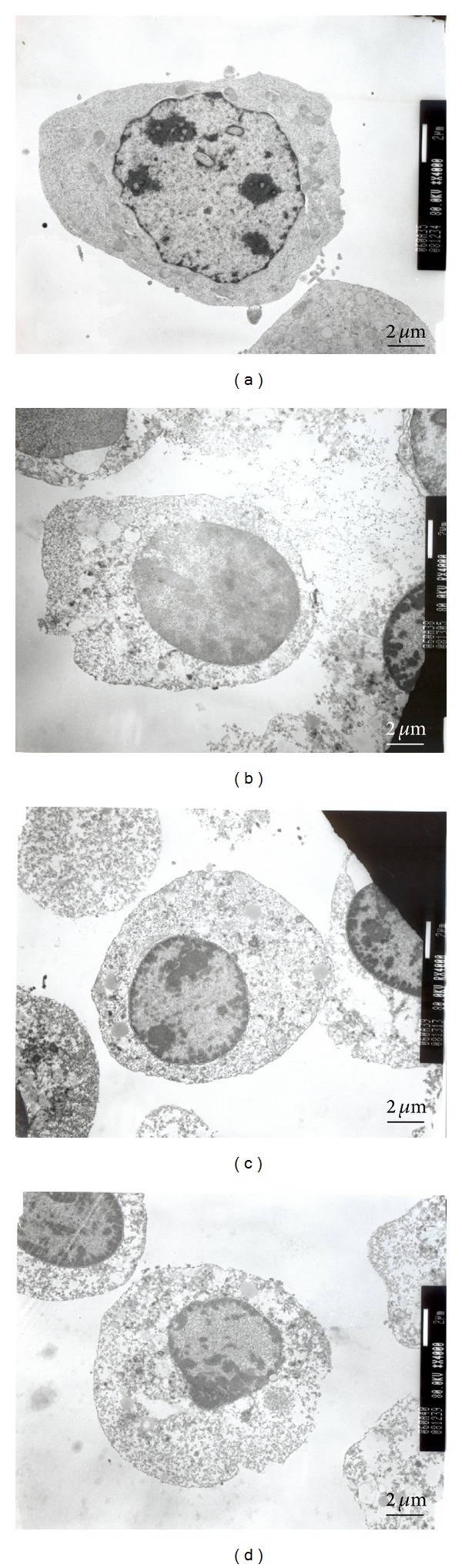
Morphological appearance of gold nanoparticles (AuNPs) with their surface modified by polyethylene glycol (PEG-AuNPs) imaged by transmission electronic microscopy. K562 cells were incubated with (a) medium only, 10 mM of PEG-AuNPs, for (b) 24 h, (c) 48 h, and (d) 72 h. Original magnification is 4,000x for all panels.

**Figure 4:**
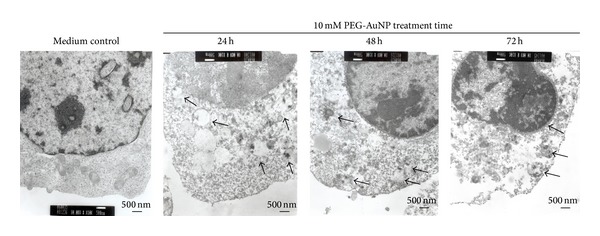
Intracellular localization of gold nanoparticles (AuNPs) with their surface modified by polyethylene glycol (PEG-AuNPs) imaged by transmission electronic microscopy. Arrows indicate the location of PEG-AuNPs. Original magnification is 10,000x for all panels.

K562 cells treated with PEG-AuNPs (10 mM for 24–72 h) were observed under a light microscope using Liu's stain. The cells showed a decrease in size after PEG-AuNPs treatment (Figure 2, middle panel), and a part of the PEG-AuNPs-treated cells showed morphological changes characteristic of apoptosis, such as membrane blebbing, fragmented nuclei, and apoptotic bodies (Figure 2, lower panel). The PEG-AuNPs-treated cells exhibited a dark red/purple color as the absorption wavelength of PEG-AuNPs is around 510–530 nm. This suggests that the cells had the ability to uptake PEG-AuNPs. To further verify the morphological appearance and localization of PEG-AuNPs, TEM was used to examine the characteristics. As shown in Figures 3 and 4, PEG-AuNPs were internalized into the cytoplasm of K562 cells at 24 h. Furthermore, PEG-AuNPs preferentially accumulated in the cytoplasmic vacuoles and disrupted the mitochondria at 48–72 h and had an increased intensity in the area surrounding the nuclear membrane at 72 h. Penetration of PEG-AuNPs into the nuclei was not observed (Figure 4).

### 3.3. PEG-AuNPs Affected Cell Cycle Distribution

**Figure 5:**
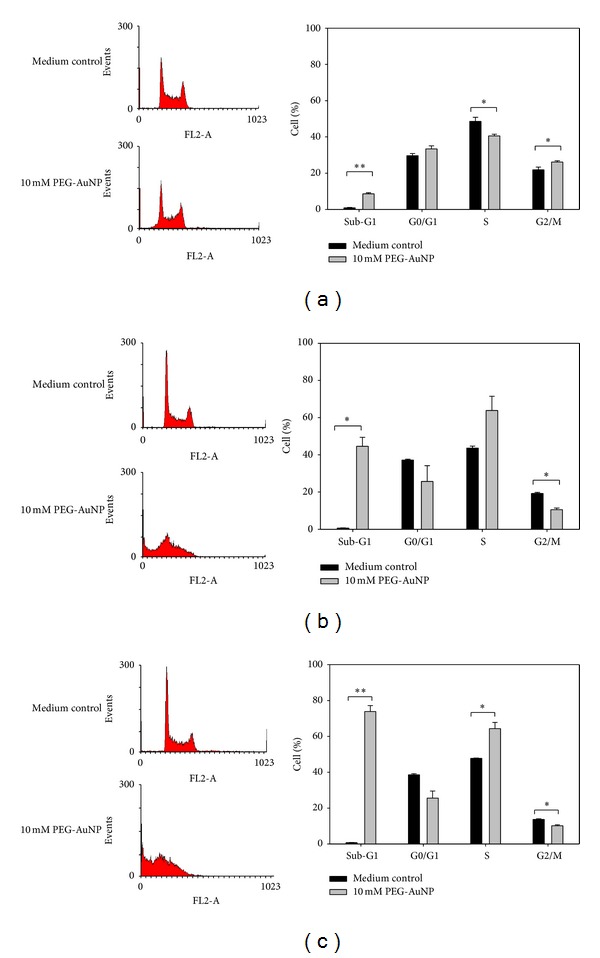
Cell cycle distribution of K562 cells treated with medium only, PEG-AuNPs 10 mM, for (a) 24 h, (b) 48 h, and (c) 72 h (compared with the control medium: *P < 0.05 and **P < 0.01). Cell cycle distribution was analyzed by flow cytometry and the percentages of cells in different cycle phases were automatically determined using ModFit cell cycle analysis software. Data are representative of three independent experiments.

To study the effect of PEG-AuNPs on cell cycle distribution, the K562 cells were treated with 10 mM of PEG-AuNPs for 24–72 h and then analyzed by flow cytometry. PEG-AuNPs treatment resulted in an increase in the number of cells in the S phase and a corresponding decrease in the number of cells in G0/G1 and G2/M phases after 48 and 72 h of treatment (Figures 5(b) and 5(c)). The percentage of sub-G1 population after PEG-AuNPs treatment increased with the incubation time (8.6 ± 0.7% for 24 h, 44.6 ± 4.9% for 48 h, and 73.9 ± 3.3% for 72 h), suggesting the induction of apoptosis (Figures 5(a), 5(b), and 5(c)).

### 3.4. PEG-AuNPs Alter Mitochondrial Transmembrane Potential in K562 Cells

To assess whether the effect of PEG-AuNPs-induced apoptosis-associated alteration in mitochondria, K562 cells were evaluated by flow cytometry to analyze the number of permeabilized cells stained with the potential-sensitive dye DiOC6_(3)_. As shown in Figure 3, the mitochondrial transmembrane potential of cells treated with 10 mM PEG-AuNPs relative to untreated controls, for 24, 48, and 72 h, were 25.0 ± 6.2%, 25.0 ± 4.5%, and 28.5 ± 3.2%, respectively. PEG-AuNPs induced a significant reduction in the mitochondrial transmembrane potential in K562 cells after 24–72 h treatment. This indicates that PEG-AuNPs may induce mitochondrial dysfunction and influence the intrinsic apoptosis pathway.

### 3.5. Fluorescence of Cells with PEG-AuNPs

It is interesting to note that PEG-AuNPs can be fluorescent at different wavelengths. In this study, fluorescence could be detected in the K562 cells treated with 10 mM of PEG-AuNPs for 24 h, with wavelengths from 500 to 700 nm by fluorescence microscopy and flow cytometry analysis (Figures 7(a) and 7(b)).

Although the fluorescence emitted from AuNPs was previously reported [20], the fluorescence is normally from much smaller nanoparticles (<2 nm), with specific surface bonded organic molecules, such as mercaptoundecanoic acid (MUA) and bovine serum albumin (BSA) [21, 22]. We noted that PEG under normal conditions is not fluorescent. Hence, the fluorescence we discovered in PEG-AuNPs could be valuable for labeling purposes. Further investigation to characterize the optical properties is underway.

## 4. Discussion

The present study demonstrated that PEG-AuNPs inhibited growth and induced apoptosis of human chronic myeloid leukemia K562 cells through the involvement of mitochondrial pathway.

Because there is considerable potential for the use of nanoparticles in medicine, the cytotoxicity as well as the cellular mechanism underlying the effect of AuNPs needs to be evaluated carefully. The cytotoxicity of the chemically synthesized AuNPs is dependent on the particle size and ligands used for surface modification. In general, the smaller the AuNPs, the higher their cytotoxicity and AuNPs conjugated with cationic ligands, such as cetyltrimethylammonium bromide, are more toxic to cells than are those conjugated with biotin, cysteine, citrate, glucose, and PEG [23–25]. In this study, approximately 6 nm diameter PEG-AuNPs were used (determined by TEM). A recent study reported by Tsai et al. used different sizes of AuNP, including small (1–3 nm), medium (5–6 nm), and large (15–20 nm), to treat K562 cells, and the reported results were similar to this study [26] because treatment with different sized AuNPs inhibited cell growth and induced apoptotic/necrotic phenotypes [26]. However, as per a study by Connor et al., neither the surface modifiers nor the size of the AuNPs exhibited cytotoxicity in K562 cells [27]. In addition, the response to PEG-AuNPs without loaded drugs may vary in different types of cancers. For example, PEGylated gold nanorods were significantly more cytotoxic to leukemia HL-60 cells than toward mammary adenocarcinoma (SKBR3), Chinese Hamster Ovary (CHO) and mouse myoblast (C2C12) cells [28].

In our study, PEG-AuNPs were internalized in the cytoplasm of the cells and accumulated in the cytoplasmic vacuoles at 24 h. (Figure 4. 24 h). Connor et al. indicated that AuNPs of different sizes and surface modifications could be internalized by the cells [27]. Similar results were also reported in the study by Krpetic et al., in which AuNPs entered K562 cells after 15 min of treatment and were located in the cytoplasm but not in the nucleus [20]. In addition, Tsai et al. reported that AuNPs were detected in various organelles of K562 cells at different times [26]. AuNPs were found inside the cytosomes, nucleus, and endoplasmic reticulum and were ubiquitous in the cytoplasm of necrotic cells at 3 h, 12 h, and 24 h, respectively [26]. Collectively, both AuNPs and PEG-AuNPs could be internalized and distributed in the cytoplasm of K562 cells.

**Figure 6:**
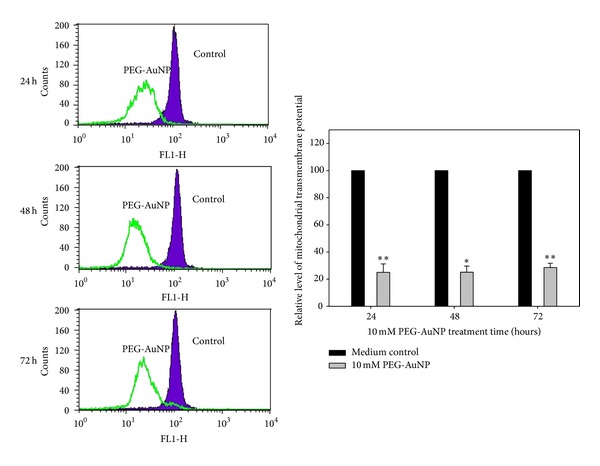
Effect of gold nanoparticles (AuNPs) with their surface modified by polyethylene glycol (PEG-AuNPs) on mitochondrial transmembrane potential reduction in K562 cells. Bar chart representing the relative level of mitochondrial transmembrane potential between K562 cells treated with PEG-AuNP and control medium for 24–72 h (compared with the control medium: *P < 0.05 and **P < 0.01).

**Figure 7:**
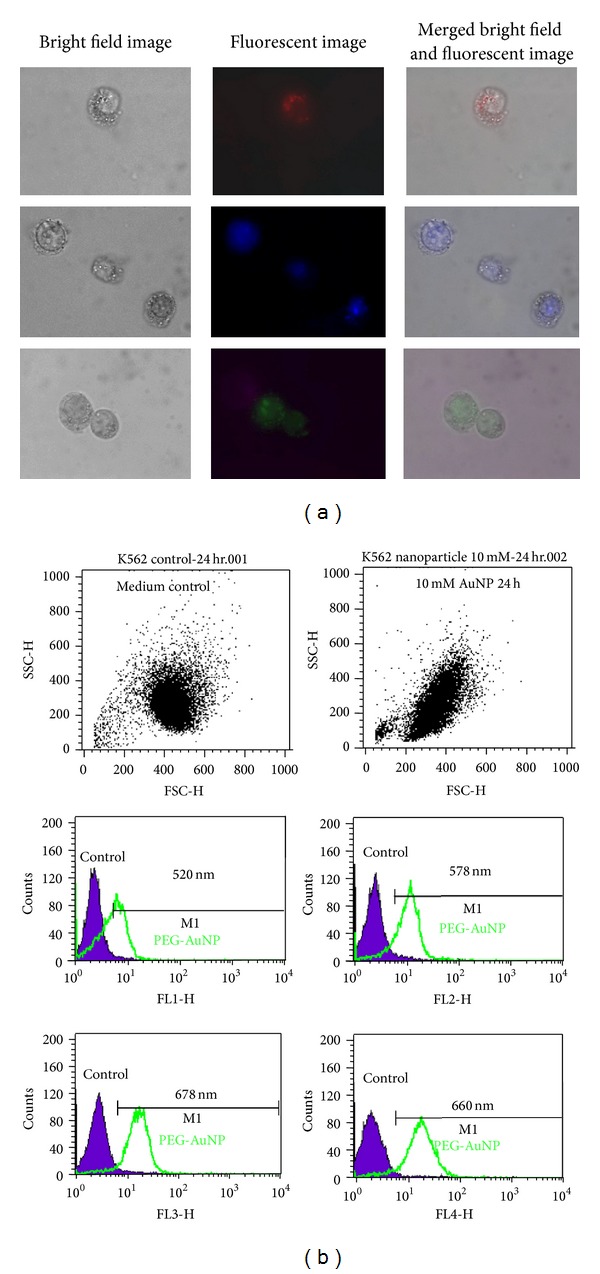
K562 cells treated with 10 mM of gold nanoparticles (AuNPs) with their surface modified by polyethylene glycol (PEG-AuNPs) for 24 h. Fluorescence could be detected with emission wavelengths from 500–700 nm by (a) fluorescence microscopy: bright field images (left), fluorescent images (middle), and merged bright field and fluorescent images (right). Original magnification is 1,000x for all panels and (b) flow cytometry analysis.

Flow cytometry analysis of K562 cells in our study showed a marked enrichment of cells in the S phase and a decrement in the G2/M phase when treated with 10 mM PEG-AuNPs for 48–72 h (Figures 5(b) and 5(c)). This suggests that the reduction in the number of cells that enter into the G2/M phase is most likely a consequence of the inhibitory effect of PEG-AuNPs on DNA synthesis in the S phase. Sub-G1 population of cells treated with PEG-AuNPs increased markedly at 24–72 h (Figures 5(a), 5(b), and 5(c)). This suggests that PEG-AuNPs induced cellular apoptosis. Mitochondria-related apoptosis was also observed as indicated by the reduced mitochondrial transmembrane potential (Figure 6). Taken together, these results indicate that PEG-AuNPs may cause mitochondrial damage and apoptosis.

PEG-AuNPs can be internalized to K562 cells and can exhibit fluorescence. These fluorescent characteristics of PEG-AuNPs may provide a novel method for tracking cancer cells.

## 5. Conclusion

To summarize, AuNPs with PEG-surface modification inhibited the growth of human chronic myeloid leukemia cells K562 and induced mitochondria-related apoptosis. In addition, PEG-AuNPs can be internalized into cancer cells and accumulated in cytosolic vacuoles. Moreover, there is potential to use PEG-AuNPs for controlling the progress of chronic myeloid leukemia, by using PEG-AuNPs as a drug delivery vehicle.
